# Supplementary material for: Multi‐dataset identification of innovative feature genes and molecular mechanisms in keratoconus
Source: J Cell Mol Med. 2024 Sep 19;28(18):e70079. doi: 10.1111/jcmm.70079 (PMC11412914; doi:10.1111/jcmm.70079)
Supplement: Supplementary file 2 — Material S2. [file JCMM-28-e70079-s002.zip › 7.GSEA analysis/3.DBNDD1/KEGG_GLYCOSPHINGOLIPID_BIOSYNTHESIS_GANGLIO_SERIES.html]

Details for gene set KEGG\_GLYCOSPHINGOLIPID\_BIOSYNTHESIS\_GANGLIO\_SERIES[GSEA]

|  || Dataset | DBNDD1.group.cls#h\_versus\_l |
| Phenotype | group.cls#h\_versus\_l |
| Upregulated in class | l |
| GeneSet | KEGG\_GLYCOSPHINGOLIPID\_BIOSYNTHESIS\_GANGLIO\_SERIES |
| Enrichment Score (ES) | -0.5705981 |
| Normalized Enrichment Score (NES) | -1.5790612 |
| Nominal p-value | 0.041666668 |
| FDR q-value | 0.697152 |
| FWER p-Value | 0.646 |
Table: GSEA Results Summary

  

Fig 1: Enrichment plot: KEGG\_GLYCOSPHINGOLIPID\_BIOSYNTHESIS\_GANGLIO\_SERIES      
 Profile of the Running ES Score & Positions of GeneSet Members on the Rank Ordered List

  

| SYMBOL | TITLE | RANK IN GENE LIST | RANK METRIC SCORE | RUNNING ES | CORE ENRICHMENT || 1 | ST3GAL5 | na | 9028 | 0.085 | -0.1940 | No |
| 2 | B3GALT4 | na | 9330 | 0.081 | -0.1400 | No |
| 3 | ST6GALNAC4 | na | 10251 | 0.068 | -0.1138 | No |
| 4 | ST6GALNAC5 | na | 11081 | 0.057 | -0.0933 | No |
| 5 | ST6GALNAC6 | na | 12319 | 0.044 | -0.0947 | No |
| 6 | HEXA | na | 13363 | 0.035 | -0.0973 | No |
| 7 | SLC33A1 | na | 14383 | 0.027 | -0.1055 | No |
| 8 | ST8SIA1 | na | 15647 | 0.018 | -0.1277 | No |
| 9 | B4GALNT1 | na | 16526 | 0.012 | -0.1434 | No |
| 10 | ST6GALNAC3 | na | 16822 | 0.011 | -0.1437 | No |
| 11 | ST3GAL1 | na | 17755 | 0.005 | -0.1669 | No |
| 12 | GLB1 | na | 18534 | -0.001 | -0.1885 | No |
| 13 | HEXB | na | 21627 | -0.028 | -0.2557 | No |
| 14 | ST3GAL2 | na | 32559 | -0.298 | -0.3394 | Yes |
| 15 | ST8SIA5 | na | 34411 | -0.518 | 0.0091 | Yes |
Table: GSEA details [plain text format]

  

Fig 2: KEGG\_GLYCOSPHINGOLIPID\_BIOSYNTHESIS\_GANGLIO\_SERIES      
 Blue-Pink O' Gram in the Space of the Analyzed GeneSet

  

Fig 3: KEGG\_GLYCOSPHINGOLIPID\_BIOSYNTHESIS\_GANGLIO\_SERIES: Random ES distribution      
 Gene set null distribution of ES for **KEGG\_GLYCOSPHINGOLIPID\_BIOSYNTHESIS\_GANGLIO\_SERIES**
